# Intracorporeal esophagojejunostomy using a linear stapler in laparoscopic total gastrectomy: comparison with circular stapling technique

**DOI:** 10.1186/s12893-020-00746-3

**Published:** 2020-05-12

**Authors:** Sejin Lee, Harim Lee, Jeong Ho Song, Seohee Choi, Minah Cho, Taeil Son, Hyoung-Il Kim, Woo Jin Hyung

**Affiliations:** 1 Department of Surgery, Yonsei University College of Medicine, 50-1 Yonsei-ro Seodaemun-gu, Seoul, 03722 Republic of Korea; 2 Gastric Cancer Center, Yonsei Cancer Center, Yonsei University Health System, Seoul, Republic of Korea

**Keywords:** Laparoscopic total gastrectomy, Esophagojejunostomy, Linear stapler, Circular stapler, Stenosis

## Abstract

**Background:**

Laparoscopic total gastrectomy for gastric cancer is feasible but less commonly performed compared to laparoscopic distal gastrectomy due to technical difficulties such as reconstruction. There is no standard esophagojejunal anastomosis technique in laparoscopic total gastrectomy due to a lack of evidence.

**Methods:**

We retrospectively analyzed data from 213 patients with gastric cancer who underwent laparoscopic total gastrectomy from October 2012 to December 2016. Of these, 109 and 104 patients underwent esophagojejunostomy with linear and circular stapling, respectively. We compared short-term postoperative outcomes, including surgical complications and anastomosis costs between both groups.

**Results:**

The mean operation time in the linear stapler group was longer than the circular stapler group (Linear stapler, 235.3 ± 57.9 vs. Circular stapler, 217.1 ± 55.8 min; *P* = 0.021); however, D2 lymph node dissection was performed more in the linear stapler group (Linear stapler, 36.7% vs. Circular stapler, 23.1%; *P* = 0.030). There were two anastomosis leakages in each group (Linear stapler, 1.8% vs. Circular stapler, 1.9%; *P* > 0.999). Anastomosis stenosis only occurred in the circular stapler group (Linear stapler, 0% vs. Circular stapler, 7.7%; *P* = 0.003). Although the linear stapling technique used more stapler cartridges (Linear stapler, 7.6 ± 1.1 vs. Circular stapler, 4.8 ± 0.9; *P* < 0.001), costs related to anastomosis were lower in the linear stapler group (Linear stapler, 1,904,679 ± 342,116 vs. Circular stapler, 2,246,150 ± 427,136KRW; *P* < 0.001).

**Conclusions:**

Esophagojejunostomy with the linear stapling technique reduces anastomosis stenosis in laparoscopic total gastrectomy. It can be recommended as a safe and more cost-effective method for esophagojejunal anastomosis.

## Background

Laparoscopic surgery for gastric cancer has become a preferred treatment option with its minimally invasive nature and benefits of short-term surgical outcomes [1]. For distal gastrectomy, ample evidence supports the technical and oncological safety of the laparoscopic approach [2–4]. Conversely, laparoscopic total gastrectomy is not commonly performed due to its technical difficulties, although the procedure is technically feasible [5–8]. Difficulties associated with esophagojejunal anastomosis and lymph node dissection along the splenic vessels are the major barriers to laparoscopy for total gastrectomy. Esophagojejunal anastomosis is closely related to surgical safety, whereas lymph node dissection is a matter of oncologic safety. The technical difficulties of esophagojejunal anastomosis make surgeons more reluctant to perform laparoscopic total gastrectomy.

Several esophagojejunal anastomosis techniques have been introduced, improving the positive initial experience for laparoscopic total gastrectomy. Initially, a circular stapling technique for esophagojejunostomy was widely employed in laparoscopic total gastrectomy since surgeons were familiar with its use during open total gastrectomy [9, 10]. The linear stapling technique was introduced more recently [11, 12]. There is no standard esophagojejunal anastomosis technique for laparoscopic total gastrectomy because only a few studies have compared laparoscopic esophagojejunostomy techniques [13–15]. Moreover, no study has compared cost-effectiveness between the linear and circular stapling techniques for esophagojejunostomy in laparoscopic total gastrectomy. We aimed to identify the optimal method by comparing postoperative outcomes, including surgical complications and cost for esophagojejunostomy for the linear and circular stapling techniques.

## Methods

### Patients

We retrospectively reviewed a prospective database of patients with gastric cancer who underwent laparoscopic total gastrectomy between October 2012 and December 2016. This study was approved by the Institutional Review Board of Severance Hospital, Yonsei University Health System (4–2016-0771), which waived the need for informed consent for the use of patient data due to the retrospective nature of the study.

A total of 213 consecutive patients underwent laparoscopic total gastrectomy with Roux-en-Y esophagojejunostomy for gastric cancer during the study period. All patients were diagnosed and evaluated preoperatively by upper endoscopy and abdominal-pelvic computed tomography. Of these, 109 and 104 patients underwent esophagojejunostomy with the linear and circular stapling technique, respectively. We collected preoperative information including age, sex, body mass index (BMI), and American Society of Anesthesiologists Physical Status from our database. We evaluated the pathological stage based on the 8th edition of the American Joint Committee on Cancer staging system.

### Surgical procedure

Surgical procedures were performed by four surgeons, who were experts with more than 200 cases of laparoscopic gastrectomy with over 3 years of experience. The anastomosis technique was determined by surgeons’ preference. Detailed procedures for stomach mobilization and lymph node dissection during laparoscopic total gastrectomy at our institution have been described previously [16].

### Esophagojejunostomy with using the linear stapler

**Fig. 1:**
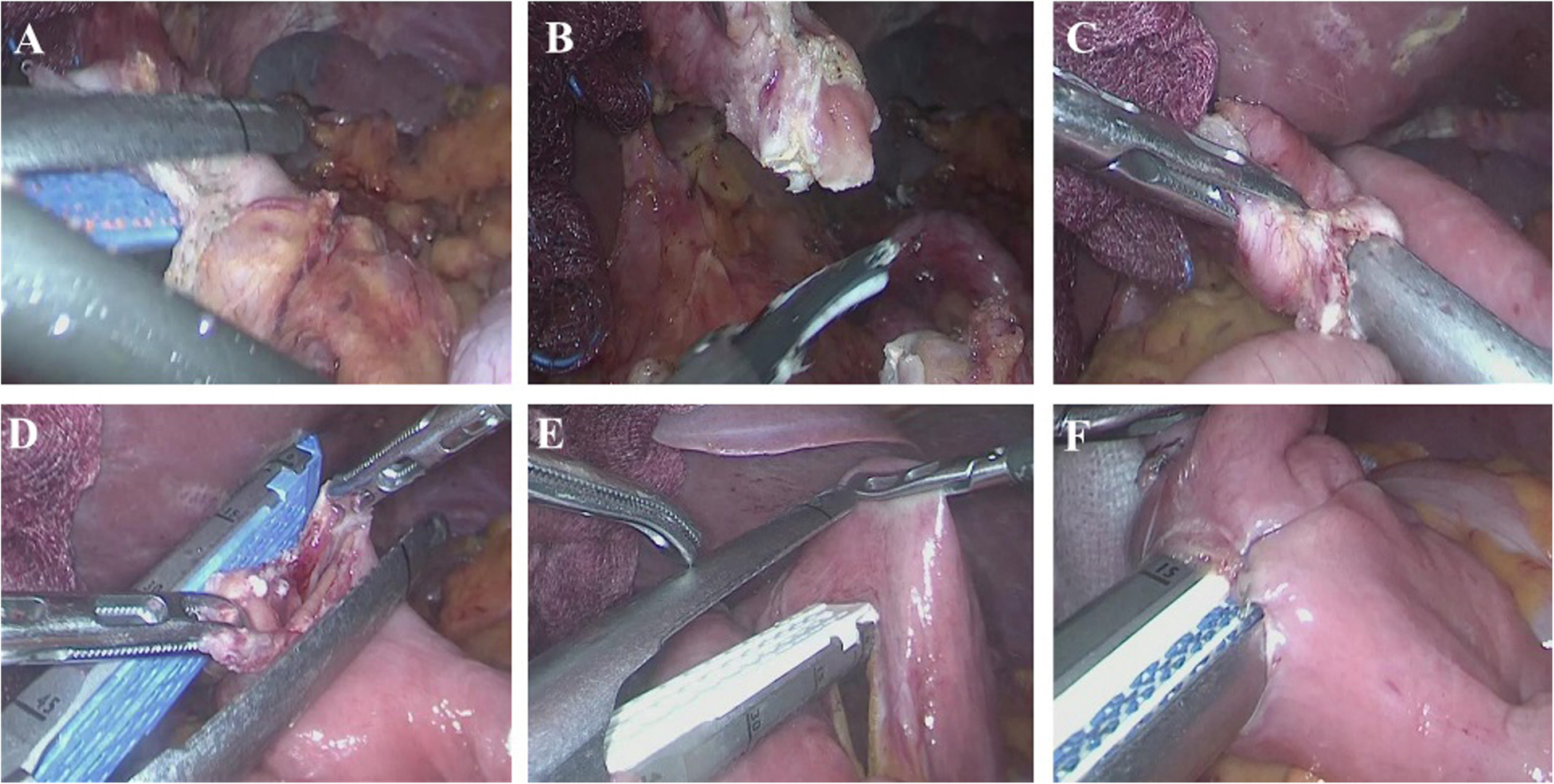
Esophagojejunostomy using the linear stapling technique. **a** Partial transection of the esophagus using a linear stapler. **b** Complete transection of the esophagus using an ultrasonic device. **c** Intracoporeal anastomosis using the overlap method. **d** Closure of the entry hole. **e** Jejunum transection without mesenteric division using a linear stapler. **f** Jejunojejunostomy 50 cm distal to the esophagojejunostomy

The exposed esophagus was adequately mobilized and rotated 90 degrees in a counter-clockwise direction to transect it from the anterior to the posterior wall. The esophagus was partially (2/3 or 4/3) transected using a linear stapler (Fig. 1a). The spared esophagus was completely transected using an ultrasonic device, leaving a small entry hole (Fig. 1b). The proximal jejunum, approximately 20–30 cm from the Treitz ligament, which is a tension-free area for anastomosis, was brought to the transected esophagus. Then, the posterior wall of the esophagus and anti-mesenteric side of the jejunum were anastomosed intracorporeally using the overlap method with a linear stapler (Fig. 1c). The common entry hole was usually closed with a linear stapler (Fig. 1d), although we occasionally performed hand-sewn closure when the anastomosis was high in the mediastinum. The biliary limb of the jejunal loop, located 2–3 cm proximal to the anastomosis, was divided without mesenteric division (Fig. 1e). Small holes were created at the antimesenteric borders of the biliary limb and the Roux limb at 50 cm distal to the esophagojejunostomy, and two loops were approximated intracorporeally by the linear stapler (Fig. 1f). The common entry hole for the jejunojunostomy was closed with a linear stapler.

### Esophagojejunostomy using the circular stapler

**Fig. 2:**
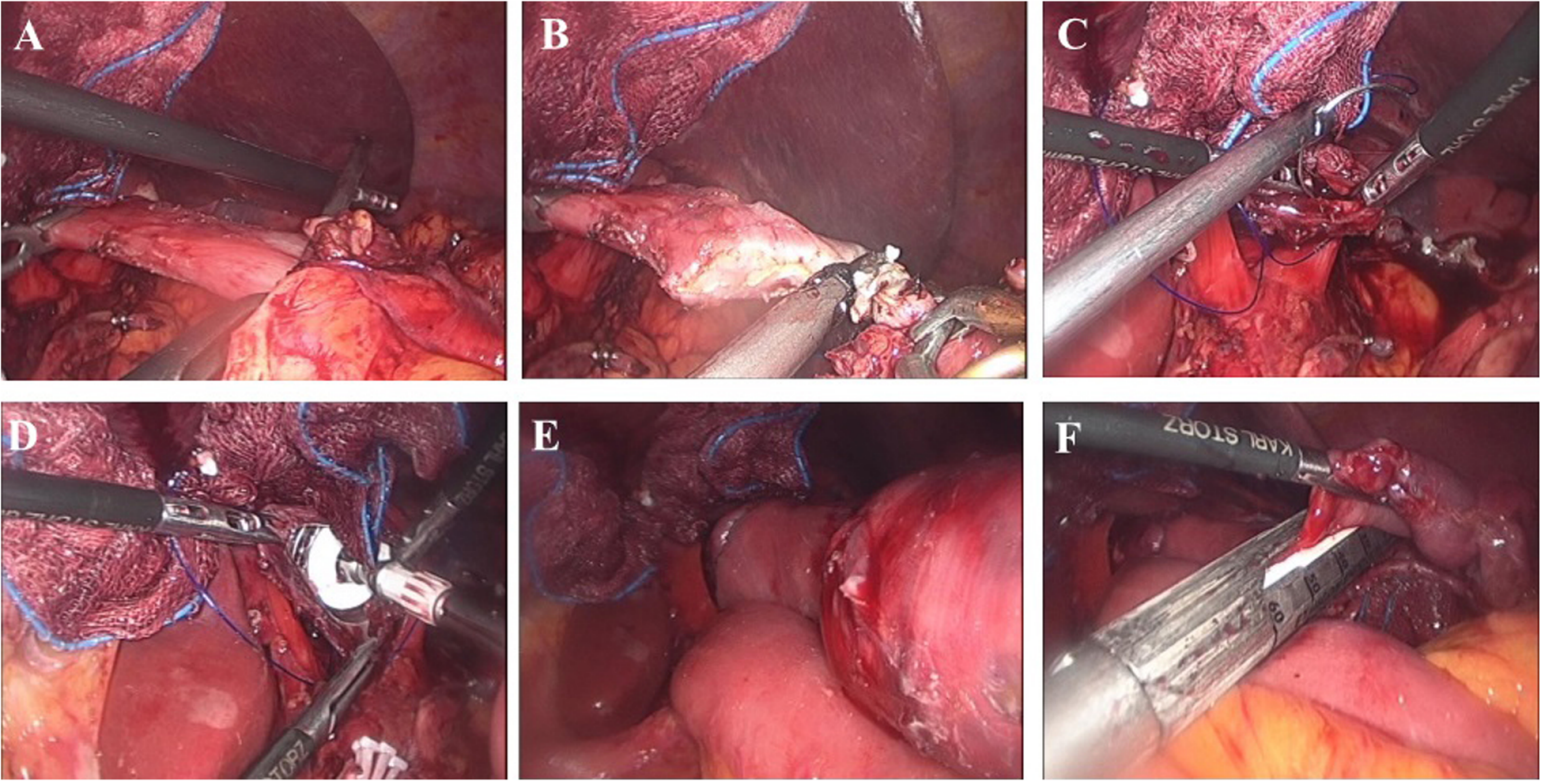
Esophagojejunostomy using the circular stapling technique. **a** Application of two laparoscopic bulldog clamps. **b** Transection of the esophagus using an ultrasonic device. **c** Purse-string suture. **d** Introduction of an anvil into the esophagus. **e** Approximation of the circular stapler. **f** Jejunal stump closure

After mobilization of the entire stomach, two laparoscopic bulldog clamps were applied on the distal esophagus using a vascular clip applicator through the right lower port (Fig. 2a). Then the esophagus was transected with an ultrasonic device (Fig. 2b). A full-layer purse-string suture was applied using non-absorbable 2–0 thickness monofilament materials (Fig. 2c). After completing the purse-string suture, the resected stomach was taken out through a 4- to 5-cm mini-laparotomy at the left lower trocar port site on the left flank. The jejunum was brought out, and a jejunojejunostomy was made extracorporeally by the linear stapler to create a side-to-side anastomosis at 50 cm distal to the esophagojejunostomy. The common entry hole for the jejunojunostomy was closed by hand-sewn or linear stapler. The anvil and circular stapler body inserted in the jejunum were introduced into the peritoneal cavity. A surgical glove with a wound protector covered the mini-laparotomy to maintain the pneumoperitoneum. The anvil was introduced into the esophagus (Fig. 2d), and the previously made purse-string suture was tied. The anvil and circular stapler body were approximated (Fig. 2e), the esophagojejunostomy was completed, and the jejunal stump was closed with a linear stapler (Fig. 2).

### Surgical outcomes, complications, and cost

We retrieved all the surgery-related variables and postoperative recovery data, including surgical outcomes and complications from our prospectively collected database. We analyzed operative time, estimated blood loss, combined resection, and extent of lymph node dissection. Time to first flatus and oral intake and postoperative hospital stay were also analyzed to assess postoperative recovery. We graded postoperative complications according to the Clavian-Dindo classification [17]. For postoperative complications, especially for evaluating anastomosis-related complications, we assessed all postoperative endoscopy and abdominal-pelvic computed tomography results for more than 2 years after surgery. We defined patients with anastomotic stenosis as treated with balloon dilatation or detected under endoscopy without dilatation. We calculated anastomosis-related costs with stapling supplies and extra supplies for all the anastomoses. The stapling supplies included the costs of the circular stapler and liner stapler body and cartridges, while the extra supplies included the costs of wound protector and suture materials.

### Statistical analysis

Statistical analyses were performed with IBM SPSS Statistics software for Windows, version 23.0 program (Armonk, NY: IBM Corp.). All data are expressed as mean ± standard deviation. Categorical variables were analyzed with chi-square and Fisher’s exact tests, and continuous variables were analyzed using Student’s t test. *P* < 0.05 was considered statistically significant.

## Results

### Clinicopathological features (Table 1)

The linear and circular stapler groups consisted of 109 and 104 patients, and their mean ages were 60.0 ± 12.0 and 58.7 ± 12.5 years, respectively (*P* = 0.440). In the linear stapler group, 63 (57.8%) patients were male compared to 72 (69.2%) in the circular stapler group (*P* = 0.083). The mean BMI in the linear stapler group was 23.5 ± 3.1 kg/m^2^ compared to 23.4 ± 2.6 kg/m^2^ in the circular stapler group (*P* = 0.687). There was no significant difference in the proportion of pathologic depth of invasion (*P* = 0.117). However, there were more patients with lymph node metastasis in the linear stapler group compared with the circular stapler group (22.0 vs. 9.6%, *P* = 0.013).

**Table 1:** Clinicopathologic features

	Linear (*n* = 109)	Circular (*n* = 104)	*p*-value
Age (year)	60.0 ± 12.0	58.7 ± 12.5	0.440
Sex			0.083
Male	63 (57.8%)	72 (69.2%)	
Female	46 (42.2%)	32 (30.8%)	
BMI (kg/m^2^)	23.5 ± 3.1	23.4 ± 2.6	0.687
ASA classification			0.108
I	17 (15.6%)	27 (26.0%)	
II	63 (57.8%)	58 (55.8%)	
III/IV	29 (26.6%)	19 (18.3%)	
pT stage			0.117
T1	73 (67.0%)	71 (68.3%)	
T2	10 (9.2%)	16 (15.4%)	
T3	16 (14.7%)	6 (5.8%)	
T4	10 (9.2%)	11 (10.6%)	
pN stage			0.013
N0	85 (78.0%)	94 (90.4%)	
N(+)	24 (22.0%)	10 (9.6%)	

*ASA* American Society of Anesthesiologists, *BMI* body mass index

### Operative outcomes and postoperative recovery (Table 2)

The mean operation time in the linear stapler group was longer than the circular stapler group (linear stapler, 235.3 ± 57.9 vs. circular stapler, 217.1 ± 55.8 min; *P* = 0.021). There was no significant difference between the two groups in mean estimated blood loss (*P* = 0.104) or the rate of combined resection (*P* = 0.323). However, D2 lymph node dissection was performed more often in the linear stapler group compared with the circular stapler group (linear stapler, 36.7% vs. circular stapler, 23.1%; *P* = 0.030).

**Table 2:** Operative outcomes and postoperative recovery

	Linear (*n* = 109)	Circular (*n* = 104)	*p*-value
Operative time (min)	235.3 ± 57.9	217.1 ± 55.8	0.021
Estimated blood loss (ml)	135.7 ± 156.1	106.6 ± 95.1	0.104
Combined resection			0.323
None	99 (90.8%)	90 (86.5%)	
Done	10 (9.2%)	14 (13.5%)	
Extent of lymph node dissection			0.030
D1 +	69 (63.3%)	80 (76.9%)	
D2	40 (36.7%)	24 (23.1%)	
Postoperative recovery			
Flatus (days)	3.3 ± 1.3	3.6 ± 1.0	0.053
SOW (days)	2.2 ± 0.5	3.7 ± 2.6	< 0.001
Soft diet (days)	4.3 ± 2.8	5.0 ± 2.8	0.073
LOS (days)	7.4 ± 5.2	7.8 ± 5.4	0.553

*LOS* length of hospital stay, *SOW* sips of water

Time to first flatus was earlier in the linear stapler group, although it was not statistically different (*P =* 0.053). Time to first water intake was significantly earlier in the linear stapler group (linear stapler, 2.2 ± 0.5 vs. circular stapler, 3.7 ± 2.6 days; *P* < 0.001); however, time to first semi-solid diet did not differ between the two groups (*P* = 0.073). The postoperative hospital stay length was not significantly different between the two groups (*P* = 0.553).

### Postoperative complications (Table 3)

Patients with postoperative complications, including major and minor complications, were 74 (67.9%) in the linear stapler group and 69 (66.3%) in the circular stapler group (*P* = 0.811). Most complications were grade I and II, including fever, wound complication, transfusion, delirium, voiding difficulty, postoperative ileus, and use of pancreas- or liver-supporting medications or antibiotics.

**Table 3:** Postoperative complications

	Linear (*n* = 109)	Circular (*n* = 104)	*p*-value
Absent	35 (32.1%)	35 (33.7%)	0.811
Present	74 (67.9%)	69 (66.3%)	
Complication gradeª			0.112
I	37 (50.0%)	33 (47.8%)	
II	29 (39.2%)	20 (29.0%)	
≥ III	8 (10.8%)	16 (23.2%)	
Type of early complications (0-30d)			
Anastomotic leakage	2	2	> 0.999
Duodenal stump leakage	1	0	0.512
Intraluminal bleeding	1	0	0.512
Intra-abdominal fluid collection	3	3	0.636
Postoperative obstruction	1	1	0.739
Pulmonary complication	3	4	0.474
Type of late complications (>30d)	3	4	0.474
Anastomotic stenosis	0	8	0.003
Postoperative obstruction	6	5	0.818
Hiatal hernia	2	0	0.498

ª Complication grade was determined according to the Clavian-Dindo classification of surgical complications

The rate of complications grade III or higher was 10.8% in the linear stapler group and 23.2% in the circular stapler group. In the linear stapler group, there were 7 (6.2%) patients with grade III complications and 1 (0.9%) with a grade V complication (0.9%). The grade III complications were pleural effusion (*n* = 1), intra-abdominal fluid collection (n = 1), and postoperative obstruction (*n* = 5), while the grade V complication was an anastomotic leakage at the esophagojejunostomy. In the circular stapler group, there were 11 (10.6%) patients with grade III complications, 4 (3.8%) with grade IV complications, and 1 (1.0%) with a grade V complication. The grade III complications were an intra-abdominal fluid collection (*n* = 1), duodenal stump leakage (*n* = 1), postoperative obstruction (*n* = 3), anastomotic leakages (*n* = 2), and anastomotic stenosis (*n* = 4); grade IV complications were pulmonary complications (*n* = 3) and postoperative bleeding (*n* = 1); and the grade V complication was postoperative bleeding.

Early complications were defined as an adverse event occurring within 30 days after surgery. Among early complications, there were 2 (1.8%) esophagojejunostomy leakages in the linear stapler group and 2 (1.9%) in the circular stapler group. All anastomotic stenosis occurred over 30 days after surgery, which regarded as a late complication in the circular stapler group (linear stapler, 0% vs. circular stapler, 7.7%, *P* = 0.003). Of these patients with anastomotic stenosis, there were 4 patients with grade III complications treated with balloon dilatation, and others had difficulty passing the endoscope, but no symptoms.

### Anastomosis-related cost (Table 4)

Total anastomosis-related cost was lower in the linear stapler group compared with the circular stapler group (linear stapler, 1,904,679 ± 342,116 vs. circular stapler, 2,250,481 ± 430,440 KRW, *P* < 0.001). Because the linear stapling technique used more linear stapler cartridges (linear stapler, 7.6 ± 1.1 vs. circular stapler, 4.8 ± 0.9, *P* < 0.001), the cost of linear stapler was higher (linear stapler, 1,871,927 ± 321,200 vs. circular stapler, 1,651,154 ± 429,688 KRW; *P* < 0.001). However, in the circular stapler group, there were the additional cost of the circular stapler (470,000 KWR) and higher extra supply costs compared with the linear stapler group (linear stapler, 32,752 ± 52,013 vs. circular stapler, 130,000 KRW; *P* < 0.001).

**Table 4:** Anastomosis-related cost

	Linear (*n* = 109)	Circular (*n* = 104)	*p*-value
Total cost^a^ (Range)	1,904,679 ± 342,116 (1,450,000-3,410,000)	2,251,154 ± 429,688 (1,660,000-3,800,000)	< 0.001
Stapling supplies cost^a^	1,871,927 ± 321,200	2,121,154 ± 429,688	< 0.001
Circular stapler	0	470,000	
Linear stapler	1,871,927 ± 321,200	1,651,154 ± 429,688	< 0.001
Number of linear stapler cartridges	7.6 ± 1.1	4.8 ± 0.9	< 0.001
Extra supplies cost^ab^	32,752 ± 52,013	130,000	< 0.001

^a^ In Korean won

^b^ Extra supplies costs included the wound protector and suture materials

## Discussion

In this study, the rates of anastomosis leakage were similar between the two groups, but stenosis of the esophagojejunostomy anastomosis was less frequent with the linear stapling technique. Furthermore, the linear stapling technique had a lower total cost for anastomosis, despite the need for more linear stapler cartridges.

Fewer stenoses in the linear stapling group were probably related to the creation of a wide lumen (> 30 mm diameter) when using 45-mm linear staplers [18]. In addition, there was less wound retraction due to an everted anastomosis at the entry hole for the linear stapling technique, resulting in less stenosis [19]. Previous studies of the linear stapling technique for the esophagojejunostomy in laparoscopic total gastrectomy also reported reductions in anastomotic stenosis compared to the circular stapling technique [20, 21].

There is no standardized assessment for esophagojejunostomy stenosis after total gastrectomy. Moreover, it is difficult to include patients who have subjective stenosis symptoms without endoscopic evidence of stenosis. Therefore, we included patients with stenosis both treated with balloon dilatation and detected under endoscopy without dilatation to avoid underestimating the incidence of stenosis.

Unlike anastomotic stenosis, there was no difference in the incidences of anastomotic leakage in our study. Previous comparative studies reported lower incidences of anastomotic leakage for the linear stapler group than the circular stapler group [14, 15]. Several factors could influence the low rate of anastomotic leakage for the linear stapling technique. It might reduce technical errors by providing a better view of the surgical field than the circular stapling technique [22]. Three rows with the linear stapler would produce a more watertight anastomosis than two rows using the circular stapler [23].

Our rates of anastomotic leakage were quite low for both stapling techniques compared to other studies [14, 15]. Similar rates are probably because we performed the same operative procedure in both groups except for using different stapler types. We preserved the jejunal vascular arcade to maintain the bidirectional arterial supply and venous drainage to prevent the ischemia and congestion at the anastomosis site, since inadequate blood supply and venous drainage are important causes of anastomotic leakage. Compared with the circular stapler, the linear stapling technique may not reduce anastomotic leakage at the esophagojejunostomy in laparoscopic total gastrectomy, provided that adequate blood supply and venous drainage at the anastomosis site are maintained.

In this study, we compared the costs of anastomosis based on the assumption that it would be more expensive to use the linear stapling technique, which uses more cartridges. Assuming the other processes are the same, the linear stapling technique during esophagojejunostomy uses two additional linear stapler cartridges than the circular stapling technique that uses a circular stapler. The cost of linear stapler cartridges varied by company (45-mm cartridge: 290,000 vs 180,000 won, 60-mm cartridge: 400,000 vs. 210,000 won, respectively). Since we typically used a 45-mm linear stapler, the price of two linear stapler cartridges is lower than that of a circular stapler (470,000 won), which requires a wound protector (130,000 won). Moreover, anastomosis cost with the linear stapling technique could be reduced if entry hole closure is done by laparoscopic suture. Therefore, the linear stapling technique is a cost-efficient anastomosis option for the esophagojejunostomy in laparoscopic total gastrectomy.

Although surgeons are familiar with using a circular stapler for esophagojejunostomy, performing this procedure laparoscopically introduces several technical complexities. In a limited laparoscopic view, it is difficult to make purse-string sutures, indwell the anvil into the esophagus, and manipulate the circular stapler [24]. In addition, a mini-laparotomy is necessary to access the esophagus during circular stapling. Mini-laparotomy is not necessary for the linear stapling technique, which conducts intracorporeal anastomosis. The linear stapler is also more comfortable to handle in a limited operative field [25]. Moreover, it can be used in patients with narrow esophagus. Based on our results, the linear stapling technique is a better esophagojejunal anastomosis method than the circular stapling technique.

Our study is limited in that we only assessed Asian patients. It is unclear if the circular stapling technique would be associated with anastomotic stenosis in Western patients who have relatively wider esophageal lumens. In our results, there were only eight patients with a BMI ≥ 25 kg/m^2^, so our findings might not be generalizable to high BMI patients that are more frequent in Western countries than Asians. From a surgical perspective, overweight patients have a higher risk of postoperative complications due to comorbidities. The surgeon’s preference in the esophagojejunostomy method and differences in the linear stapler cartridge length and stapler manufacturers are additional limitations. A well-designed randomized controlled trial with a large, heterogeneous cohort is required to identify the optimal anastomosis method for esophagojejunostomy in laparoscopic total gastrectomy.

## Conclusion

In laparoscopic total gastrectomy, esophagojejunostomy by the linear stapling technique can reduce anastomosis stenosis compared to the circular stapling technique. Linear stapling can be recommended as a safe and more cost-effective option for esophagojejunal anastomosis.

## Data Availability

The data that support the conclusion of this study are available from the corresponding author upon reasonable request.

## Abbreviations

BMI: Body mass index
ASA: American Society of Anesthesiologists
LOS: Length of hospital stay
SOW: Sips of water
